# The efficacy of bosentan combined with vardenafil in the treatment of postoperative pulmonary hypertension in children with congenital heart disease: A protocol of randomized controlled trial

**DOI:** 10.1097/MD.0000000000023896

**Published:** 2021-01-08

**Authors:** Chao Gao, Junting Liu, Runhan Zhang, Manting Zhao, Yongli Wu

**Affiliations:** Cangzhou Central Hospital, Cangzhou, Hebei Province, China.

**Keywords:** bosentan, combination therapy, congenital heart disease, pulmonary hypertension, vardenafil

## Abstract

**Background:**

Congenital heart disease in children with pulmonary hypertension is a common and serious complication, which has a direct impact on the surgical effect and prognosis of children. Bosentan and vardenafil are commonly used drugs for the treatment of postoperative pulmonary hypertension in children with congenital heart disease, and there are few clinical studies on their combined use. Therefore, the purpose of this randomized controlled trial is to evaluate the effectiveness and safety of the combined use of 2 drugs in the treatment of postoperative pulmonary hypertension in children with congenital heart disease.

**Methods:**

This is a prospective randomized controlled trial to study the effectiveness and safety of bosentan combined with vardenafil in the treatment of postoperative pulmonary hypertension in children with congenital heart disease. Approved by the clinical research ethics committee of our hospital. The patients were randomly divided into 1 of 2 treatment regimens:

1. bosentan combined with vardenafil oral group and
2. vardenafil oral group.

Patients, doctors, nurses, and data collection assistants were blinded to group allocation. Observation indicators include: oxyhemoglobin saturation (SpO2), 6-min Walking Test Distance (6 MWTD), systolic pulmonary artery pressure, mean pulmonary artery pressure, Borg score, NYHAFC score, etc. Data were analyzed using the statistical software package SPSS version 25.0 (Chicago, IL).

**Discussion:**

This study will evaluate the effectiveness and safety of bosentan combined with vardenafil in the treatment of pulmonary hypertension after congenital heart disease in children. The results of this experiment will provide a clinical basis for the use of bosentan combined with vardenafil to treat pulmonary hypertension after congenital heart disease in children.

**Ethics and dissemination:**

Private information from individuals will not be published. This systematic review also does not involve endangering participant rights. Ethical approval was not required. The results may be published in a peer-reviewed journal or disseminated at relevant conferences.

**OSF Registration number:**

DOI 10.17605/OSF.IO/962BT.

## 1 Introduction

Congenital heart disease (CHD) refers to vascular or heart malformations caused by abnormal growth of cardiac blood vessels during the embryonic period. It is a common cardiovascular disease in pediatrics,^[1]^ due to the large amount of left to right shunts at the defect site of CHD. The blood volume of the pulmonary circulation increases significantly, and the high flow puts the pulmonary blood vessels in a state of high pressure and high resistance, causing pulmonary arterial hypertension. Pulmonary hypertension is one of the common and serious complications of congenital heart disease in children. About 10% of children with congenital heart disease develop into PAH,^[2]^ which can occur at various stages in the evolution of congenital heart disease in children. The surgical effect and prognosis of patients have a direct impact, and the perioperative mortality rate of children with CHD and severe PAH is higher.^[3]^ Therefore, effectively reducing pulmonary hypertension is a key step to improve the surgical efficacy and quality of life of CHD patients.

There are currently 3 types of targeted therapy drugs recommended, including endothelin receptor antagonists, prostaglandin i2, and phosphodiesterase 5 inhibitors (PDE-5 inhibitors).^[4]^ Bosentan is a representative drug of endothelin receptor antagonists, which has the effects of reducing pulmonary vascular resistance, improving hemodynamic indicators, improving exercise tolerance, and improving the patient's survival rates.^[5,6]^ Vardenafil is a highly selective inhibitor of phosphodiesterase-5 (PDE-5), which can selectively inhibit PDE-5 activity and reduce the degradation of cyclic guanosine monophosphate (cGMP). The latter can further activate protein kinase G, increase potassium ion channels, reduce calcium ion concentration, thereby reducing pulmonary artery pressure. At present, clinical studies have pointed out that the two-drug combination therapy can significantly improve the patient's exercise capacity, improve the patient's heart function and relieve the symptoms of dyspnea,^[7]^ but there are too few similar studies, and the sample size is not large, and it is difficult to draw reliable conclusions. Therefore, the purpose of this randomized controlled trial is to evaluate the effectiveness and safety of bosentan combined with vardenafil in the treatment of postoperative pulmonary hypertension in children with congenital heart disease.

## 2 Materials and methods

### 2.1 Study design

**Figure 1:**
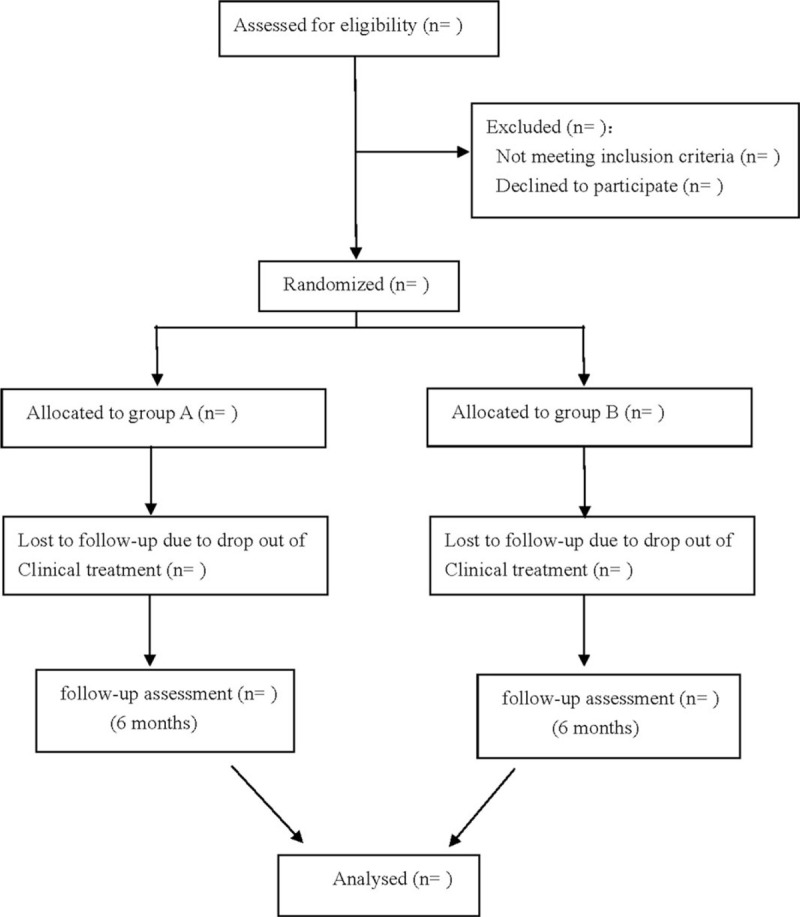
Flow diagram.

This is a prospective randomized controlled trial to study the effectiveness and safety of bosentan combined with vardenafil in the treatment of postoperative pulmonary hypertension in children with congenital heart disease. We followed the Consolidated Standards of Reporting Trials (CONSORT) guidelines for reporting randomized trials and provided a CONSORT flow diagram (Fig. 1) and the Standard Protocol Items: Recommendations for Interventional Trials (SPIRIT) 2013 statement.

### 2.2 Ethics and registration

This research program is in accordance with the Helsinki Declaration and approved by the Clinical Research Ethics Committee of our hospital. This protocol has been registered in open Science Framework (OSF) (Registration number: DOI 10.17605/OSF.IO/962BT). All patients need to sign a written informed consent before they are randomly assigned to continue the trial.

### 2.3 Sample size

The calculation of the sample size is based on the results of the 6 MWTD. According to the results of the pre-test, the average score of the experimental group is estimated to be 495.47 and the standard deviation is 54.72. The control group averaged 456.33 and the standard deviation was 54.68. The sample size calculation formula is as follows

$$n=n1=n2=\frac{{\left(u_{\alpha }+u_{\beta }\right)}^{2}\times \sigma^{2}}{\delta^{2}}\times 2$$

At the 5% significance level, a total of 34 patients are required for each group to achieve 90% power. The estimated withdrawal rate is 20%, and 42 patients will be included in each group.

### 2.4 Patients

Inclusion criteria:

1. All patients meet the diagnostic criteria of congenital heart disease-pulmonary hypertension (CHD-PAH),^[8]^ aged> 6 months and <16 years old;
2. There is a risk of reactive pulmonary hypertension or pulmonary hypertension at the end of the operation Phenomenon;
3. postoperative pulmonary artery systolic pressure/systemic arterial systolic pressure (Pp/Ps) ≥0.3, no decrease within 1 week;
4. patients with moderate or higher pulmonary hypertension found in postoperative follow-up;
5. preoperative pulmonary vascular resistance index (PVRI) ≥4 persons.

Exclusion criteria:

1. Children with other types of pulmonary hypertension related to non-congenital heart disease;
2. Patients with liver and kidney dysfunction;
3. Patients with prolonged QT syndrome;
4. Patients concurrently using antiarrhythmic drugs;
5. Allergic to test drugs;
6. Severe digestive system or blood system abnormalities;
7. Unable to understand after explanation.

The research protocol may be unwilling to participate.

### 2.5 Study design

Eligible participants were randomly assigned to the treatment group or control group at a ratio of 1:1 using a random tool based on the central network. Randomization was performed without any stratification using SAS 9.3 software (SAS Institute, Cary, NC, USA) by independent statisticians who were not involved in the implementation of the experiment or statistical analysis. Randomization was performed without any stratification. The clinical research coordinator enters participant information on the tablet and is given a random number. The research assistant gets the participant's assignment from the computer. Throughout the research process, the research assistant is responsible for screening, recruiting participants, and assigning random numbers to the included participants. The result assessor is responsible for the assessment of the scale. All bed doctors, researchers, research assistants, participants, intervention supervisors, and statisticians who perform statistical analysis are unknowable in the grouping of personnel.

The dispensation of medicines will be assigned to patients with corresponding numbers by unknowing nurses according to the medicine numbers.

### 2.6 Intervention

Control group: Oral vardenafil hydrochloride tablets, 20 mg/time, 1 time/d;

Treatment group: Oral bosentan tablets on the basis of the control group, according to the weight of the patient, weight <10 kg: 15.625 mg/time; weight: 10 to 20 kg: 31.25 mg/time; weight: 21 to 40 kg: 62.5 mg/time, 1 time/d.

The treatment lasts for 6 months. During the treatment period, the patients will be followed up by outpatient or telephone in the first and third months of treatment to understand the symptoms and adverse reactions of the patients, and review the observation indicators at the sixth month.

### 2.7 Observation index

1. Oxyhemoglobin saturation SpO2, using GEM3000 blood gas analyzer to measure SpO2 before and after treatment in the 2 groups;
2. (2) 6-min walking test distance (6 MWTD): For children who can cooperate with walking to walk back and forth in a quiet and airy 30-m corridor, measure the 6-min walking distance;
3. The patient uses a SwanGanz floating catheter for right heart catheterization in a resting state to determine systolic pulmonary artery pressure, pulmonary artery pressure, and aortic pressure. Fick method is used to calculate mean pulmonary artery pressure, pulmonary circulation blood flow quantity/systemic circulation Blood flow quantity, pulmonary artery systolic pressure/systemic artery systolic pressure;
4. The patient got 5 ml of fasting venous blood in the morning before and after treatment, and the brain natriuretic peptide was measured by enzyme-linked immunosorbent assay;
5. Borg score^[9]^ (divided into 0–10 grades, 10 is the most serious, consciously exhausted), cardiac function classification (NYHAFC);^[10]^
6. Adverse reactions: including dizziness, headache, facial flushing, abnormal liver, and kidney function, etc.

### 2.8 Data collection and management

One or 2 assistants will collect and record the entire data. Personal information about potential participants and registered participants will be collected, shared and stored in an independent storage room to protect confidentiality before, during and after the test. The access to the database will be restricted to the researchers in this study team.

### 2.9 Statistical analysis

Data were analyzed using the statistical software package SPSS version 25.0 (Chicago, IL). Continuous variables were described as the mean ± standard deviation, and differences between groups were analyzed using a series of one-way analysis of variance (ANOVA) with Bonferroni posthoc test, while differences between groups over time were analyzed using multiway ANOVA with Bonferroni posthoc test. Categorical variables were described as the number (%), and were analyzed by Fisher exact test. A *P* value of <.05 was considered statistically significant.

## 3 Discussion

Pulmonary hypertension is one of the common complications after congenital heart disease, and it is also an important risk factor for death from right heart failure.^[11]^ With continuous research on the pathogenesis of pulmonary hypertension, it has been found that vasoconstriction, cell proliferation, inflammation, fibrosis, and thrombosis are all related to pulmonary vascular remodeling, while thromboxane, exhaled nitric oxide (eNO), and endothelin-1 (ET-1) and prostaglandin and other vasoactive mediators play an important role in it.^[12,13]^ Therefore, how to effectively reduce the patient's pulmonary vascular resistance and contain or reverse pulmonary vascular disease is of great significance for improving the survival rate of patients and improving the quality of life.

Endothelin (ET) is an active substance with the strongest vasoconstrictor effect. The main place for its production and removal is lung tissue, which is one of the important factors that cause pulmonary hypertension. Bosentan tablets are competitive antagonists of endothelin ETA and ETB receptors. They can compete to antagonize human vascular wall ET-1 receptors, inhibit vasoconstriction and promote cell proliferation,^[14]^ and can reduce lung and systemic vascular resistance, increase cardiac output without increasing heart rate.^[15]^ Vardenafil is another new PDE-5 inhibitor after sildenafil, which can significantly reduce arterial pressure (PA) and venous pressure at the same time.^[16]^ In addition to inhibiting PDE-5 and passing In addition to the nitric oxide-cyclic guanosine monophosphate(NO-cGMP) dependent mechanism that causes pulmonary artery relaxation, it can also activate some mechanisms that do not depend on NO-cGMP: nitric oxide-cyclic guanosine monophosphate, that is, induce pulmonary vasodilation by inhibiting the entry of extracellular Ca^2+^.^[17,18]^

Some studies have pointed out that bosentan combined with vardenafil or sildenafil is better than monotherapy in the treatment of pulmonary hypertension after congenital heart disease in children,^[7,19]^ in 2015 the European Society of Cardiology (ESC) pulmonary artery. The guidelines for the diagnosis and treatment of hypertension recommend starting oral drug combination therapy for patients with cardiac function class II to III.^[20]^ The combination therapy is becoming more and more important for the treatment of pulmonary hypertension after children with congenital heart disease. This study will be evaluated the effect of bosentan combined with vardena on the treatment of pulmonary hypertension after nonchildren's congenital heart disease.

This study also has the following limitations: Since this is a singlecenter randomized controlled study, the included population may be regionalized, and the results may be biased; factors such as the age of the patients included in the study may have an impact on the results.

## Author contributions

**Data curation:** Chao Gao and Junting Liu.

**Investigation:** Junting Liu and Runhan Zhang.

**Resources:** Manting Zhao.

**Funding acquisition:** Yongli Wu.

**Software:** Runhan Zhang.

**Supervision:** Yongli Wu.

**Writing – original draft:** Chao Gao and Junting Liu.

**Writing – review & editing:** Chao Gao and Yongli Wu.
